# The microenvironment of brain metastases from solid tumors

**DOI:** 10.1093/noajnl/vdab121

**Published:** 2021-11-27

**Authors:** Ethan S Srinivasan, Krutika Deshpande, Josh Neman, Frank Winkler, Mustafa Khasraw

**Affiliations:** 1 Duke Brain and Spine Metastases Center, Duke University, Durham, North Carolina, USA; 2 Department of Neurological Surgery, Keck School of Medicine, University of Southern California, Los Angeles, California, USA; 3 Department of Neurological Surgery, Physiology and Neuroscience, USC Brain Tumor Center, Norris Comprehensive Cancer Center, Keck School of Medicine, University of Southern California, Los Angeles, California, USA; 4 Neurology Clinic and National Center for Tumor Diseases, University Hospital Heidelberg, Heidelberg, Germany; 5 Clinical Cooperation Unit Neurooncology, German Cancer Consortium, German Cancer Research Center, Heidelberg, Germany

**Keywords:** brain metastasis, immune suppression, microenvironment, neural niche, therapeutic targets

## Abstract

Brain metastasis (BrM) is an area of unmet medical need that poses unique therapeutic challenges and heralds a dismal prognosis. The intracranial tumor microenvironment (TME) presents several challenges, including the therapy-resistant blood–brain barrier, a unique immune milieu, distinct intercellular interactions, and specific metabolic conditions, that are responsible for treatment failures and poor clinical outcomes. There is a complex interplay between malignant cells that metastasize to the central nervous system (CNS) and the native TME. Cancer cells take advantage of vascular, neuronal, immune, and anatomical vulnerabilities to proliferate with mechanisms specific to the CNS. In this review, we discuss unique aspects of the TME in the context of brain metastases and pathways through which the TME may hold the key to the discovery of new and effective therapies for patients with BrM.

The importance of dynamic interactions between the native tissue components and metastatic tumor cells has become increasingly apparent over the past few decades of cancer research. When metastatic cells invade, they integrate into and modify the environment in complex pathways with important implications to tumor establishment, aggressiveness, and response to treatment. Understanding these relationships within the tumor microenvironment (TME) is essential to the exploration of brain metastasis (BrM) research and clinical progress. Today, BrMs comprise over 50% of all intracranial tumors and occur in up to 40% of all patients suffering from metastatic cancer.^1–4^ The incidence of clinically relevant BrM is likely to further increase with improved treatment of primary tumors, and the relative paucity of specifically approved treatments highlights the importance of innovation. In all cases, the intricate web of interactions between metastatic cells and the microenvironment of the central nervous system (CNS) begins before any actual seeding and evolves in a temporally and spatially dependent manner. Eluding to the complexity of the brain’s microenvironment, we now have a better understanding from patient spatial modeling that there is a nonuniform spatial distribution of metastasis to preferential brain regions according to primary cancer subtype. Use of predictive spatial modeling to reveal that primary cancers have distinct CNS topography patterns of BrM.^5^ These cancer-specific brain topography patterns may underlie the ability of tumor cells to adapt to regional neural microenvironments in order to facilitate colonization and establishment of metastasis. While the full range of interactions might be heterogeneous across tumor origins and mechanisms, there are key shared elements and pathways that can be considered to better understand the current state of the field, its future, and the implications for clinical advancements in the treatment of this particularly irascible disease

Even before consideration of BrM, the CNS microenvironment is a unique compartment within the body. The resident cells create a complex and dynamic microenvironment in their own rights, with interactions between neurons, astrocytes, oligodendrocytes, microglia, pericytes, immune cells, and the extracellular matrix essential to normal development and function. This ecosystem is separated from the peripheral vasculature by the blood–brain barrier (BBB), a selective filter composed of tightly connected endothelial cells, pericytes, and astrocyte projections within a dense basement membrane. The invasion of metastatic cells into this closely regulated environment results in an evolving TME distinct from any other seen in systemic metastases. In this review, we will cover how this distinct environment governs metastatic seeding and growth in the brain, and how this knowledge can be used to develop improved clinical concepts to treat or to prevent BrM. We start with the various microenvironmental factors of the brain metastatic cascade, then focus on 4 microenvironmental aspects that we consider key for a better understanding of fundamental BrM biology, and that at the same time are also instructive for clinical translation. These are the perivascular niche, angiogenesis and blood vessel interactions, tumor–immune interactions, and neuron–tumor crosstalk.

## Premetastatic Niche

**Figure 1.**
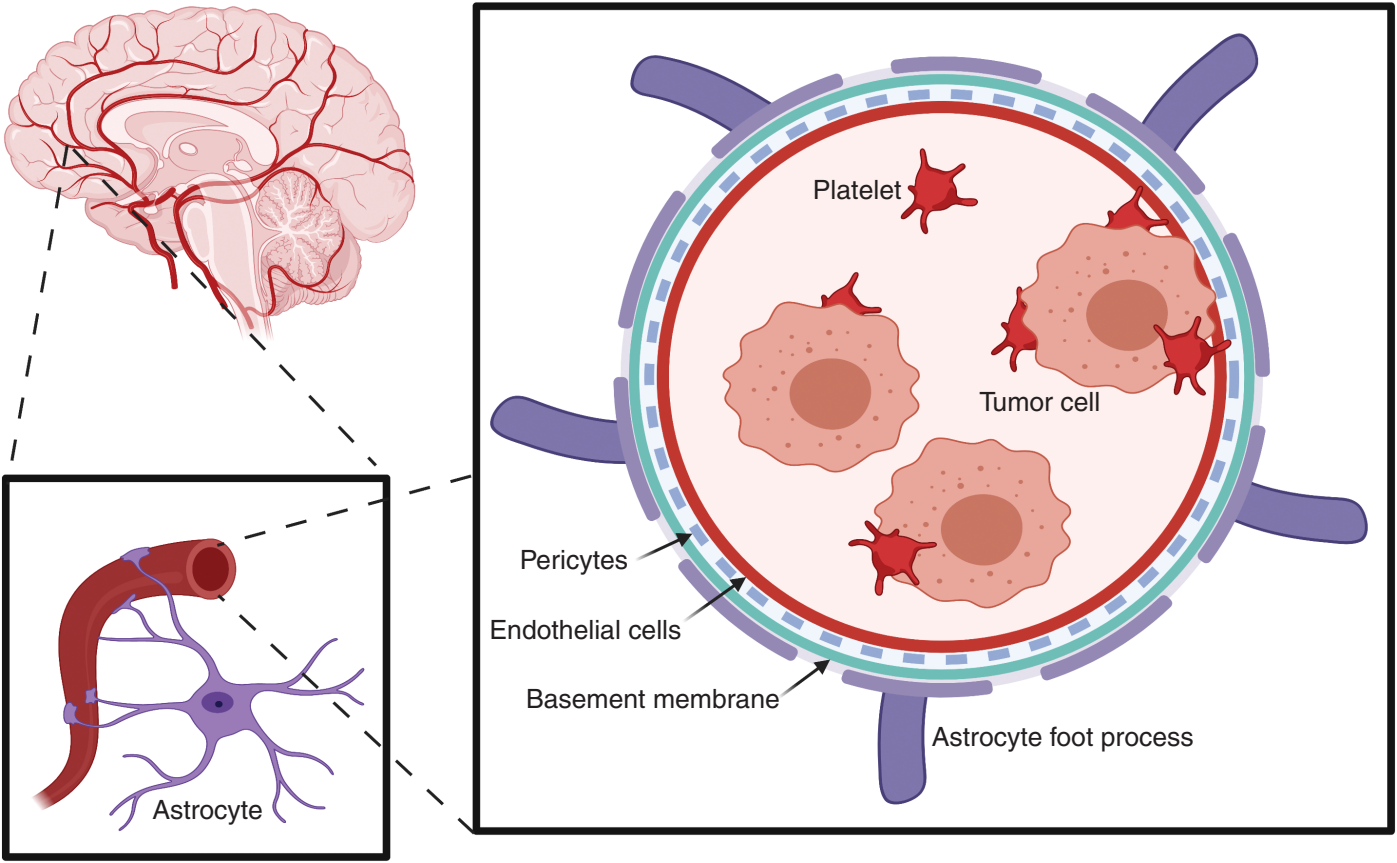
Diagram depicting blood–brain barrier organization and platelet-supported arrest of circulating tumor cell within the cerebral microvasculature. The figure was created with biorender.com.

Prior to extravasation of metastatic cells through the BBB into the CNS environment (Figure 1), it is possible that there is an establishment of a permissive environment in the brain. This phenomenon is often termed the “premetastatic niche” and is the result of secreted cytokines, chemokines, exosomes, and angiogenic factors from the primary tumor location and circulating tumor cells. Unfortunately, it has not been as well characterized in the specific case of solid tumor BrM as in other systemic locations, and therefore a discussion of this necessitates some degree of extrapolation and inference. A wide range of factors has been identified in metastases to other sites, including TNF-α, TGF-β, and vascular endothelial growth factor (VEGF), among many others.^6–8,9(p1),10(p2),11^ One unique element of the process to the CNS is the initial attack on the BBB. Feng et al.^12^ utilized a murine model of acute monocytic leukemia to show that BBB permeability was increased by secreted matrix metalloproteinases 2 and 9, through disruption of the tight junctions (TJs) between endothelial cells. This functional change is the result of the downregulation of essential TJ proteins ZO-1, claudin-5, and occludin.^12^ The same group further characterized the role of secreted VEGF in compromising the BBB through similar mechanisms, and subsequent work has shown an ameliorating effect with VEGF inhibition via monoclonal antibody.^13,14^ The work by Li et al.^15^ in small cell lung cancer found that BrM is more common in patients with high levels of placental growth factor (PLGF). Their study of the effect in vitro revealed that the secreted factor also acts through a VEGFR-mediated pathway to break down TJs, exposing the underlying CNS to invasion by circulating tumor cells. Furthermore, Lyle et al.^16^ identified alterations in the pericyte population of high-permeability BBB metastases, showing an enriched population of desmin + pericytes. Beyond undermining the structural integrity of the BBB, expression of tumor-specific cellular adhesion molecules is also increased prior to metastatic colonization. In a murine breast cancer model, Soto et al.^17^ found that the brain endothelial vascular cells upregulate multiple cell adhesion molecules (CAMs), in particular, E-selectin, vascular cell adhesion molecule (VCAM-1), ALCAM, ICAM-1, VLA-4, and β4-integrin, in response to circulating tumor cells. At the same time, these molecules were upregulated on the vascular surface, their ligands were similarly highly expressed on the circulating tumor cells both, highlighting a potential mechanism for increased susceptibility to CNS colonization.

The CNS microenvironment beyond the BBB is also modulated by circulating factors prior to metastasis. Morad et al.^18^ highlighted the influence of tumor-derived exosomes, finding that such complexes containing proteins, nucleic acids, and lipids in a breast cancer model can enter the CNS parenchyma through the BBB by compromising the endothelial cell endocytic pathway. Fong et al.^19^ identified a role for these exosomes with their study on complexes containing the miR-122 miRNA, which suppress glucose uptake in astrocytes and create a more hospitable environment for nascent metastatic cells. In their in vivo model of breast cancer BrM, the group showed that inhibition of miR-122 recapitulated normal glucose uptake in the brain and decreased the incidence of metastasis. Another implicated miRNA is miR-181c, which has been also demonstrated to disrupt the BBB and increase the incidence of BrM in an in vivo breast cancer model, in this case through the disruption of an actin localization pathway via downregulation of the PDPK1 gene.^20^ Rodrigues et al.^21^ identified an exosome-contained factor, CEMIP, that promotes metastatic invasion of the CNS. This protein was found to be specifically upregulated in brain-tropic metastatic cells and induces pro-metastatic changes in both brain endothelial cells and microglia by supporting a pro-inflammatory milieu. The group added further evidence to their results with the finding that high CEMIP expression in patients’ primary tumor samples correlated to a shorter time to BrM and worse overall survival. Altogether, the development of a premetastatic niche by secreted factors from the primary tumor is an important step in establishing a pro-metastatic microenvironment at the future site of metastasis.

## The Tumor Cell of Origin for Brain Metastases and Its Road to the Brain

Tumor cells are continuously circulating in the bloodstream over the course of cancer growth, but only a small fraction will eventually grow to a clinically relevant brain macrometastasis. This is certainly partly due to the inefficiency of the brain metastatic cascade after tumor cell arrest and extravasation in the brain. In light of extensive tumor cell heterogeneity in all adult cancer entities studied so far, it is likely that only a tiny subfraction of tumor cells can “seed.” Recent work has shown that BrM-initiating breast cancer cells are particularly slow-cycling and express various stemness pathways.^22^ This confirms earlier studies that demonstrated a slow-cycling phenotype of breast cancer circulating tumor cells (CTCs) in the clinical setting of brain metastatic disease and a particular brain metastatic potential of cancer cells with a more stem-like phenotype.^23–25^ It is unknown whether we can molecularly better characterize brain metastasis-initiating cancer cells in the future and develop more effective approaches for their specific targeting for the prevention of BrM.

It is widely accepted that BrM is hematogenous, which means that CTCs in the bloodstream arrive in brain (micro)vessels, arrest, and extravasate. This is supported by extensive intravital imaging studies and also the fact that no anatomical system exists in humans that would explain a lymphogenic arrival in the brain.^26^ However, the finding that leukemic cells from the bone marrow use small bridging veins (emissary vessels) with laminin-rich microenvironments to reach the CNS raises the question whether similar mechanisms can also be in place for CNS colonization of solid malignancies, which frequently disseminate to the bone marrow, too.^27,28^ Malignant cells may travel along the abluminal surface of vessels that are topologically contiguous with the subarachnoid space, and these cells can migrate directly to the CNS, bypassing the need to enter and exit the vasculature.^29^ This anatomic trafficking pathway has also recently been shown to play a role in inflammatory processes, suggesting the possibility of an important function in immune surveillance and CNS tumor responses.

Intriguingly, breast cancer patients who develop leptomeningeal metastases do not always have parenchymal metastases, but they all have vertebral bone metastases.^30,31^ These clinical observations may suggest that distinct molecular programs underlie brain parenchymal versus meningeal metastasis.

## Tumor Microenvironment

### Perivascular Niche

The initial intravascular arrest, extravasation, and initial colonization of the CNS by circulating tumor cells mark the next step in the evolution of the brain TME. Even the intravascular arrest is associated with relevant microenvironmental changes in the brain: microthrombosis and reduced microvascular perfusion, which likely impact the microenvironment. Platelet clotting and fibrin formation at the very site of arrested tumor cells were important for their long-term persistence and extravasation in the brain, with multiple interesting avenues for BrM prevention particularly by anticoagulatory therapies.^32^ The exact cellular process of extravasation in the brain is not well understood so far, but appears to be a dynamic process of tumor cell–blood vessel wall interactions.^26^ After tumor cell extravasation that is already mastered by a minority of brain-arrested cancer cells only, the following metastatic cascade into the CNS occurs across a harsh selective pressure, with fewer and fewer cells progressing to the next step of the sequence. Kienast et al.^26^ characterized the first foray of metastatic cells beyond the BBB with real-time in vivo multiphoton laser-scanning microscopy study of BrM formation. The vast majority of even the extravasated cancer cells fail to establish a foothold within the tissue, and the surviving cells remain near the vessel wall in the critical perivascular niche. Whenever they leave that niche in the first 2 weeks after brain colonization, they die. Studies in primary brain tumors have revealed the lasting importance of this location within the tumor, as a key site for the cancer stem cells that drive tumor growth and angiogenesis through a range of mechanisms including differentiation into vascular endothelial cells.^33–35^ In the initial stages, the perivascular niche is the crow’s nest from which the proliferating tumor cells can redirect vascular remodeling to meet their own needs. In glioblastomas, the perivascular niche also serves as a cancer stem cell reservoir that is critical to tumor progression and treatment resistance.^36–39^ Translationally, the German consortium (*prevent*_BM) has focused on key biological processes in this crucial perivascular niche for survival and resistance in the brain to develop novel molecular therapies against the earlier steps of BrM for improved BrM prevention.

**Figure 2.**
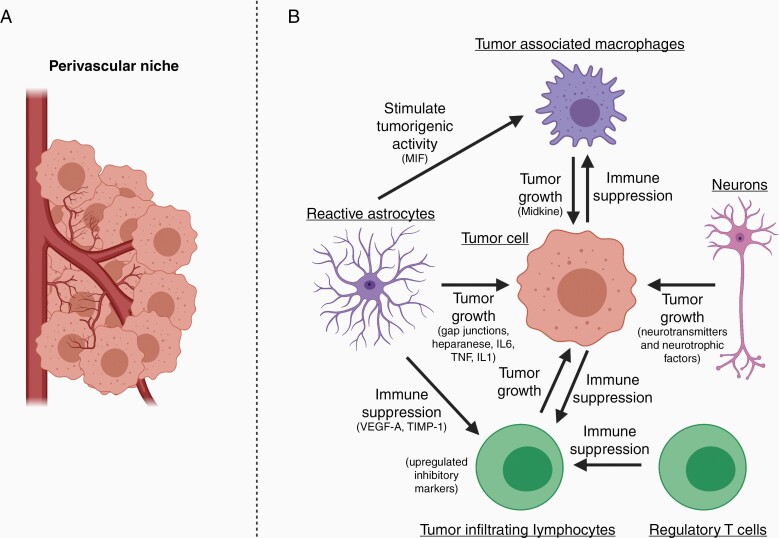
(A) Diagram depicting vascular co-option and VEGF-mediated angiogenesis within the perivascular niche of the tumor microenvironment. (B) Summary of major interactions within the tumor microenvironment of brain metastases between tumor cells, tumor-associated macrophages, reactive astrocytes, neurons, and tumor-infiltrating lymphocytes. The figure was created with biorender.com.

This reorganization of the cerebral microvasculature is an essential step in the metastases’ progression. Vascular appropriation by the growing metastatic lesion proceeds through 2 primary pathways, first through co-option of existing vasculature and second through stimulation of angiogenesis.^26^ The degree to which each pathway is present varies by tumor type.^26,40,41^ In vascular co-option (Figure 2), the tumor cells take advantage of the existing structures to obtain essential nutrients and oxygen for proliferation. This process is dependent on several adhesion molecules including L1CAM and β1-integrin that mediate interactions with the vascular basement membrane.^42,43^ Loss of these mechanisms was further demonstrated to attenuate metastatic proliferation, highlighting the importance of vascular co-option particularly in early colonization.^42^ Beyond simply anchoring, the interactions between the vascular basement and metastatic tumor cells induce proliferative and invasive profiles, as shown by Er et al.^41^ through YAP and MRTF signaling, that lead to spreading throughout the perivascular niche. A further study highlighting the differences between metastatic and primary sites from Jubb et al.^44^ found that matched BrM in non-small cell lung cancer were significantly more likely to depend on tumor co-option rather than angiogenesis. This difference points both to the selective pressures of the metastatic cascade and important differences that are clinically relevant when considering therapies targeted towards these pathways.

### Angiogenesis

Angiogenesis can provide a modified niche for perivascular cancer cells, but its implications extend beyond that aspect. Regarding tumor-directed angiogenesis, the primary driving mechanism appears through the VEGF pathway. On its own, VEGF stimulates angiogenesis and increased vascular permeability.^45^ Tumor cells directly secrete the factor into their surrounding environment to stimulate the growth of complex microvascular networks, along with its activation through other mediators including integrin-α _v_β _3_.^40,46,47^ Anti-VEGF therapy has shown interesting results thus far, reducing angiogenesis, preventing an early angiogenic switch, and preventing and reducing metastatic growth in preclinical models, along with angiopoietin-2 inhibition (see the Clinical Implications section for more details).^26,47–50^

### Tumor Cell Entry Into the Brain: Barriers of the CNS

The brain is structurally and functionally isolated from the peripheral circulation, to limit exposure of the CNS to external influences at interfaces where the blood and the cerebrospinal fluid (CSF) interact with the neural milieu. To this effect, the brain has evolved to have 2 main barriers of restriction, namely, the BBB and the blood cerebrospinal fluid barrier (BCSFB).^51^ Current research indicates that tumor cell entry into the brain occurs mainly via crossing of the BBB. However, recent data suggest the involvement of BCSFB as a route of entry for circulating tumor cells that reside within the CSF. Although these barriers tightly regulate neural homeostasis and immune privilege in the CNS, their restrictive nature also contributes to obstacles for effective drug delivery into the brain.^52^ Thus, tumor cells that successfully cross into the brain parenchyma use the brain as a sanctuary from chemotherapeutic insult and immune clearance.

The BBB consists of a non-fenestrated capillary endothelium, ensheathed by a network of pericytes, astrocytic foot processes, and microglia that together constitute this neurovascular unit. Central to its barrier functions are tight and adherens junctions that regulate the selective permissiveness of the BBB and restrict paracellular diffusion between plasma and brain interstitium. TJs connect adjacent endothelial cells and consist of 3 membrane proteins, namely, claudin, occludin, and junction adhesion molecules. These are further associated with cytoplasmic accessory proteins like Zona occludens (ZO-1,2,3) and cingulins which are anchored to the actin cytoskeleton. Adherens junctions comprise of cadherin–catenin complex and associated proteins.^53^

Although burdened with the critical function of protecting the brain against injury, inflammation, and pathogens, the integrity of the BBB is compromised in conditions like stroke, brain trauma, and neurodegenerative diseases like multiple sclerosis and Alzheimer’s disease.^54^ Similarly, brain-seeking tumor cells that arrest within the BBB capillaries can alter the barrier properties, enabling entry into the brain parenchyma. Extravasation into the brain takes longer than in other organs, and studies show it takes lung cancer (LC) cells 48 hours, breast cancer 2–7 days, and melanoma up to 14 days to invade the BBB.^26,55^ Enhanced migratory capacity of tumor cells within the BBB vasculature and interference in normal endothelial structure and function contribute to CNS metastasis. BrM from triple-negative and basal breast cancer is associated with BBB disruption based on GLUT1 and BRCP expression profiles, while Her2^+^ BrM tends to preserve BBB function.^56^ Melanoma cells in vitro induce endothelial apoptosis and reduce trans-endothelial resistance by proteolytic disruption of TJs claudin-5 and ZO-1 during migration, after which the endothelium repairs itself.^57^ These cells also interact with VCAM-1 found on the surface of endothelial cells activated by stimuli like TNF-α and interferons, indicating that tumor cells extravasate the BBB preferentially at sites of inflammation. PDX models show that the BBB is disrupted in BrM from breast, lung, and prostate cancers due to downregulation of Msfd2a, a fatty acid transporter expressed in the endothelium.^58^ This is accompanied by impaired TGFb and FGF signaling in the endothelium and loss of normal astrocyte function within the BBB.^59^ The expression of alpha2,6-sialyltransferase ST6GALNAC5, usually restricted to the brain, was found to be enhanced in BrM breast cancer cells, enhancing their adhesion to the endothelial cells and passage through the BBB.^60^ In LC, tumor-derived PLGF and enhanced expression of proteases MMP9 and ADAM10 facilitate passage through the BBB through disassembly of TJs in VEGFR1 expressing endothelial cells, and degradation of BBB ECM.^15,61^ Breast cancer cells also secrete exosomes containing miRNAs 105 and 181c and transfer these miRNAs to endothelial cells, disrupting BBB TJ functionality.^20^ Thus, although there is heterogeneity in the mechanisms of BBB disruption, brain-seeking tumor cells collectively modulate the brain endothelium to attempt successful BrM colonization.

CTCs in the CSF and leptomeninges are therapeutic roadblocks and potential “seeds” of metastasis in the brain and spine.^23^ The CSF comes into close contact with blood in 2 areas, which formally make up the BCSFB: (1) where the arachnoid membrane envelopes the subarachnoid space and (2) where choroid plexus (CP) projects into the ventricular system.^62^ Because CP microvasculature lacks TJs, it is porous to large molecules, unlike the BBB endothelium. This is due to the absence of capillary ensheathment by astrocytic foot processes and the expression of “pore-forming” claudin-1 in choroid plexus, rather than “barrier-forming” claudins 3, 5, and 12 evident in the brain. The functional BCSFB is therefore dependent on TJs within choroid epithelium, rather than their “leaky” endothelium. Correspondingly, the BCSF is more permeable with a TEER of 150 ohm cm^2^ in vitro, compared to 1500 ohm cm^2^ measured in the BBB in vivo.^63^

Despite its contact with the CSF and its high permeability, the BCSFB is an understudied route of tumor cell entry into the CNS. Rare cases of intraventricular metastases have been reported from renal, lung, GI, breast, and bladder cancers, some found to be juxtaposed right alongside CP cells that line the lateral ventricles.^64^ Increase in tumor-derived complement C3 within the CSF was shown to disrupt the BCSFB by activating the C3a receptor in the choroid epithelium. This leads to unrestricted inflow of growth factors and nutrients across the BCSFB, facilitating the growth of leptomeningeal metastases.^65^ Recent in vitro studies show that while LC cells migrate through the BBB and BCSFB at comparable rates, breast cancer cells preferentially migrate through the BCSFB by degrading TJs in the choroid epithelium.^66^ Neuroblastoma cells were able to migrate across an intact BCSFB in a paracellular fashion within 24 hours in vitro, without affecting the integrity of choroid epithelium.^67^

### Metabolic Niche of the Microenvironment

Metabolomics is a rapidly developing field within the study of oncology broadly with significant relevance to the specific and general cases of the metastatic microenvironment. The brain itself is a hub of metabolism within the body, consuming 20% of available glucose-derived energy along with its ability to rapidly adapt to various metabolic states and alternative energy sources, including acetate, glutamine, and branched-chain amino acids.^68^ Studies have demonstrated that the CNS microenvironment imposes specific and distinct metabolic pressures on metastatic tumors, as similar energy source flexibility has been found in brain metastases relative to matched primary tumors.^69,70^ Fischer et al.^71^ identified in their study of melanoma brain metastases an increased utilization of oxidative phosphorylation pathways compared to paired extracranial metastases. These findings offer direct translational opportunities, as they demonstrated in vivo efficacy of an oxidative phosphorylation inhibitor on murine survival. In a separate study, the same group showed that greater enrichment of oxidative phosphorylation expression is clinically relevant and associated with shorter survival after resection in humans, which could be targeted in their mouse model through metformin treatment.^72^ Fukumura et al.^73^ supported these findings with their 2021 study that found enhanced oxidative phosphorylation across lung, breast, and renal cell carcinomas again compared to matched primary or extracranial metastatic tumors, mediated through a separate mechanism from the PGC-1α implicated in melanoma.^74^ In breast cancer, Ebright et al.^75^ identified elevated HIF1A expression compared to matched primaries, a mediator of hypoxic signaling associated with glycolytic pathways. Ngo et al.^76^ highlighted a similar effect with their findings of the importance of PHGDH, a catalyst in the serine synthesis pathway, that provides metastatic cells with an essential amino acid of limited availability within the CNS. Additionally, specific cell populations within the CNS have been implicated in driving the metabolic profile of brain metastases. Zou et al. demonstrated that interactions between astrocytes and melanoma brain metastases activate PPARγ within the metastatic tumor, which is critical to modulation of glucose homeostasis and fat metabolism, with a resultant sensitivity to PPARγ inhibitors specific to CNS metastases. The authors implicated the lipid-enriched brain microenvironment and specifically polyunsaturated fatty acids within astrocytes in the mechanism of this effect.^7^ These results emphasize a theme of distinct but parallel pathways leading to common endpoints within the CNS microenvironment. The ability of metastatic cells to overcome the resource limitations of the brain is an essential predictor of their success, and this condition presents an additional dimension for specific targeting of CNS metastases.

### Interaction With Cells of the CNS Microenvironmental Niche

#### Astrocytes

Astrocytes are glial cells involved in tissue homeostasis, maintenance of the BBB, regulation of neuronal synaptic responses, and immune signaling.^77^ Owing to their diverse functionality, they also play important roles in the disease progression of CNS malignancies. In BrM, astrocytes exhibit both pro- and antitumor functions.^78^ Early after tumor cell infiltration, astrocytes are activated by gliosis and contribute to neuro-protection by inducing tumor cell death through nitric oxide production and plasmin secretion, and forming reactive astrocyte (RA) boundaries delineating the metastatic lesion from the adjacent normal brain.^43,79,80^ However, they also promote BrM formation and colonization at various stages. They protect tumor cells from chemotherapy, and support BrM growth through the formation of tumor–astrocyte gap junctions that inhibit intratumoral calcium uptake, and stimulate release of tumor-supportive cytokines through the innate STING pathway.^81,82^ In vitro studies show enhanced tumor cell growth on co-culture with astrocytes. These effects are attributed to the release of soluble factors like heparanase, IL6, TNF, and IL1 by astrocytes, which stimulate metastatic determinants like endothelin-1 receptor expression, ERK phosphorylation, and induction of survival genes like *BCL2L1* and *TWIST1* in tumor cells.^80,83–86^ Astrocytes also regulate immune response within the brain during BrM progression. Priego et al.^87^ identified the presence of STAT3^+^ RAs in established BrM. The secretome from these RAs was sufficient to suppress T-cell activation and limit any antitumor effects on BrM cells through immunosuppressive molecules like VEGF-A, TIMP-1. Furthermore, they demonstrated that in response to macrophage migration inhibitory factor secretion from these RAs, CD74^+^ macrophages in the BM vicinity express midkine, a factor that promotes tumor cell growth.

### Neurons

Neurons mediate the propagation of electro-chemical signals within the brain and are critical for the normal functioning of the CNS milieu. However, our understanding of their role in BrM progression is limited. BrM lesions cause solid stress on surrounding brain tissue resulting in neuronal loss and neurological dysfunction.^88^ RAs and microglia also contribute to neuronal death through persistent inflammation in response to tumor cell exposure. Furthermore, unlike normal breast epithelium, metastatic breast cancer cells express low levels of the serpin pigment epithelium-derived factor (PEDF) contributing to reduced neuronal health during BrM progression. Restoration of PEDF in xenografted mice results in increased tumor suppression.^89^

The seed and soil hypothesis states that successful BrM can be established by tumor cells that can adapt to the neural niche. Recent studies have highlighted the gain of neuro-adaptive attributes in brain-seeking tumor cells, particularly in breast cancer. Neman et al.^90^ showed that BrM breast cancer cells shift their metabolic requirements and adapt GABAergic properties usually attributed to neurons. Breast cancer cells also show enhanced expression of receptors for BDNF and NGF, 2 major neurotrophic factors indicating the ability to respond to these stimuli within the neuronal niche.^91^ Neurons, heretofore assumed to be bystanders in BrM, are now shown to be directly involved in tumor cell growth. Aggressive breast cancer cells upregulate GluN2B, a metabotropic glutamate receptor, that primes them for successful BrM. Within the brain, these cells form pseudo-tripartite synapses with surrounding astrocytes and neurons and utilize glutamate secreted by presynaptic neurons, augmenting tumor growth and BrM colonization.^92^

#### Tumor-associated macrophages

The CNS has parenchyma-associated resident innate immune cells called microglia and nonparenchymal macrophages in the perivascular, meningeal, and choroid plexus regions.^93^ Our knowledge of the heterogeneity and function of brain macrophages in the progression of CNS metastasis is limited. CD68^+^ or Iba1^+^ brain macrophages were detected within and around BrM lesions from breast and lung cancer and melanoma. Although these tumor-associated macrophages (TAMs) showed markers of phagocytosis, they did not seem to activate inflammation or adaptive immunity, indicating adaptation of a pro-tumorigenic phenotype.^94^ In vivo studies show accumulation of activated and reactive brain-resident microglia and infiltrating macrophages within BrM lesions, leaning toward a pro-tumorigenic subtype particularly in parenchymal BrM compared to those in the dura.^95^ In vitro, microglia promote breast cancer cell growth and invasion in co-culture, as well in living brain tissue slices. These properties could be blocked by inhibition of microglia function with bisphosphonate clodronate or by Wnt antagonist DKK-2.^96^ Currently, our knowledge of TAM involvement in leptomeningeal metastases is limited; however, perivascular and meningeal macrophages may play a role at these sites due to their role in immune cell recruitment to the CNS.^97^

## Other Tumor-Infiltrating Immune Cells

The presence of infiltrating immune cells into the TME of BrM presents both an additional challenge and a well of potential therapeutic targets. Friebel et al.^98^ demonstrated through their single-cell mass cytometry study of resected tumors that BrM has a greater proportion of invading immune cells relative to primary brain tumors. The effector CD8+ T cells were found to express high levels of both co-stimulatory and co-inhibitory molecules. Furthermore, they showed a preferential accumulation of regulatory T cells (Tregs) in BrM relative to gliomas. Typically these cells serve an essential role in tempering auto-immunity; however, in the setting of the metastatic brain TME, their anti-inflammatory activity creates a more permissive environment for tumor progression.^99^ The immunosuppressive environment of brain tumors has been highlighted in both primary and metastatic tumors.^100^ Analysis of BrM samples from a range of primary tumor types revealed distinct trends across histologies regarding distribution and phenotyping. Harter et al. identified melanomas and renal cell carcinomas as the most immunologically active tumors, with diffuse infiltration throughout melanoma samples, though infiltration has been shown in a range of histological origins.^101,102^ In this study as well, a high degree of inhibitory markers was identified, highlighting the problem of tumor-induced T-cell dysfunction.^102^ Ogiya et al.^103^ conducted a study of matched tumor samples from primary and metastatic brain tumors in breast cancer patients and found significantly lower infiltration of immune cells in the BrM. Completion of similar studies across other primary tumor types will be important to characterizing heterogeneity within the metastatic microenvironment and its implication for future treatment.

Song et al.^104^ observed that peripheral T cells may not require access to the brain, because they can experience the parenchymal antigenic repertoire within the deep cervical lymph nodes (dCLNs). In contrast to glioblastoma, combined anti-PD1 and anti-CTLA4 have intra- and extracranial activity in melanoma. Mice with either intracranial or flank tumors benefit from checkpoint inhibitor therapy while survival benefit in mice with only intracranial tumors treated with the VEGF-C (a growth factor for lymphatic vessels) with an anti-PD1 combination therapy were similar to mice with both intracranial and flank tumors treated with checkpoint inhibitor therapy alone. Ligation of the dCLNs removed the VEGF-C benefit in mice with intracranial tumors, but not mice with both intracranial and flank implants. T-cell priming through expression of VEGF-C in the CSF, or through a flank tumor, enables checkpoint inhibition in the CNS. However, in the case of a tumor that is confined to the CNS at a steady state (eg, glioblastoma), immune checkpoint inhibitors alone do not confer notable benefits. Educating immune cells outside the CNS may hold the key to a new strategy to increase lymphatic drainage, thereby enhancing immunosurveillance and overcoming the immune ignorance of CNS tumors.

Beyond infiltrating lymphocytes, neutrophils have also been found to be highly abundant within BrM. These cells have been shown to be similarly influenced by the TME, with upregulation of anti-inflammatory markers including ADORA2A.^105^ Additionally, these cells exhibit a phenotype that actively inhibits local T-cell proliferation, further emphasizing the co-option of immune effector cells into a pro-tumorigenic microenvironment by BrM.^105,106^ The prognostic implications for the degree of immune infiltration are currently unclear, with some studies indicating no correlation and others linking infiltrating immune cell density to both edema and overall survival.^102,107^ Beyond local immunosuppression, brain tumors are also linked to systemic lymphopenia and exhaustion.^108^ With these populations of cells both present within the TME and systemically available, identifying therapeutics to functionally activate them is an important pursuit going forward.

## Clinical Implications

Measures taken in clinical practice for prevention of BrM include prophylactic whole-brain radiotherapy (WBRT) in tumors with a high risk of CNS relapse like small cell lung cancer, although it is only offered to a minority of patients because of potential neurotoxicities. New more targeted and well-tolerated approaches are needed.

The preventive efficacy of antiangiogenic drugs appears to be restricted to a limited number of tumor types (eg, lung adenocarcinoma) and the brain, which provides an example of how the brain TME can differ from the rest of the body.^26,50,109^ In addition to monoclonal antibodies targeting VEGF-A, tyrosine kinase inhibitor, nanobody targeting VEGF-A and Angiopoietin-2, have also shown activity in BrM, which indicates a class effect of these drugs.^109^ Clinically, the anti-VEGF-A antibody bevacizumab can exert even single-agent effects in BrM of breast, lung, and colorectal cancers, as shown in a recent case series and earlier studies.^110–112^ However, response assessment using MRI can in principle be misleading in this setting as antiangiogenic therapies can reduce gadolinium uptake, although this seems much less the case than in glioma.^48^ However, these anti-VEGF-A treatment effects in BrM patients appear clinically meaningful, particularly as a salvage therapy for heavily pretreated patients, and may also mitigate the need for immunosuppressive corticosteroids.

Standard treatment of BrM is typically centered on radiotherapy, with surgical resection indicated in cases with significant mass effect or hydrocephalus. Today, stereotactic radiosurgery (SRS) has largely replaced WBRT, particularly in cases with limited metastatic loads.^113,114^ Unfortunately, historically BrMs are known to be particularly treatment-resistant to both systemic and radiotherapeutic approaches. SRS has been shown to extend survival in these cases, though melanoma and renal cell carcinoma are known to be particularly radioresistant.^115^ Tumor resistance to systemic therapy is often attributed to obstruction and active exclusion by the BBB, in particular, p-glycoprotein (ABCB1) mediated efflux.^116–118^ However, interactions within the TME have also been shown to play important roles in BrM chemoresistance. RAs within the tumor drive upregulation of survival genes in tumor cells, in particular, GSTA5, BCL2L1, and TWIST1. Kim et al.^86^ demonstrated that upregulation of these genes was directly correlated with tumor resistance, and that their regulation was directly dependent on gap junction communication between astrocytes and tumor cells, with disruption increasing therapeutic efficacy.

Additional mechanisms of chemotherapy resistance have been shown to be mediated by signals within the brain TME, along with calcium sequestration by RAs.^82,119^ Interactions within the microenvironment have been shown to increase treatment resistance through the PI3K–AKT–mTOR pathway and PTEN loss within metastatic cells.^119–122^ Importantly for consideration of future therapeutics, inhibition across these pathways increased the susceptibility of tumor cells to traditionally ineffective chemotherapy agents.^120^ Various small molecular inhibitors targeting driver mutations including HER2, EGFR, ABL, and BRAF have shown efficacy in the treatment of appropriately selected BrM; however, broad success has not been found as most tumors do not have targetable oncogenes.^123–125^

Attempts to reverse the immunosuppressive environment of BrM to harness the circulating and infiltrating immune cells have been an active area of research. Essential key questions to future investigations are highlighted in Table 1. Thus far, successes have been primarily limited to the most immunologically active cancer subtypes. Melanoma and non-small cell lung cancer have seen particular clinical efficacy, with benefits to immunotherapy targeting the PD1 pathway in initial smaller studies.^126,127^ Kluger et al.^128^ administered pembrolizumab, an inhibitor of the PD1 pathway, in 23 patients with asymptomatic melanoma BrM and identified a promising overall survival rate of 48% at 2 years. Another study targeting the CTLA4 pathway in 72 patients showed activity against melanoma BrM along with systemic responses, also with evidence of long-term survivors (26% overall survival at the 2-year timepoint).^129^ Knisely et al.^130^ found clinical benefits with the addition of ipilimumab, an anti-CTLA4 agent, to SRS in their study of 77 patients. In a subsequent study by Amaral et al.^127^ in 380 patients, a corresponding benefit was found for the combination of immunotherapy, using both PD-L1 and CTLA4 inhibition, with surgery and radiosurgery upfront. These findings highlight the concept of combinatorial therapies and the likelihood that utilizing several methods to broadly and specifically target tumors is a compelling avenue forward in the treatment of BrM. Clinical trials of immunotherapeutics in BrM are ongoing, which will provide important evidence of their role and applications in future multimodal treatment paradigms.

**Table 1.** Essential Key Questions for Future Research on the Tumor Microenvironment of Brain Metastases

Gaps in Knowledge and Areas of Future Research - Which neurotransmitters/neuroreceptors are relevant for the brain metastatic process, and for which step (early brain colonization vs macro-tumor growth), beyond NDMAR? - What is the most promising future concept for *prevention* of brain metastasis? For this, we need to understand the crucial, rate-limiting cellular and molecular steps of brain metastasis better. However, several vulnerabilities of cancer cells and their brain metastatic process have already been discovered, and temozolomide, anticoagulants, and PI3K/mTOR inhibitors in very low, well-tolerated dosing appear current promising candidates for current or future clinical brain metastasis prevention trials.^32,131,132^ - How can we improve the duration of immunotherapies in the brain? - What are the molecular characteristics of the circulating cancer cell of origin for the brain metastatic process, and how can it be exploited? - How does intrapatient tumor heterogeneity compare in brain vs nonbrain metastases? What is the impact of therapy? How can we measure intrapatient heterogeneity better?

It is clear today that the TME in BrM is of high and specific pathobiological importance and at the same time provides ample avenues for more effective therapies. The emerging data from neuron–cancer cell interactions, angiogenesis, brain’s barrier, specific immunological features, and other aspects that are covered in this review hold the promise to extend our armamentarium to treat or even prevent this challenging disease.

## References

[1] Ostrom QT, Chen Y, M de Blank P, et al The descriptive epidemiology of atypical teratoid/rhabdoid tumors in the United States, 2001–2010. Neuro Oncol. 2014;16(10):1392–1399. 24847086 10.1093/neuonc/nou090 PMC4165422

[2] Barnholtz-Sloan JS, Sloan AE, Davis FG, Vigneau FD, Lai P, Sawaya RE. Incidence proportions of brain metastases in patients diagnosed (1973 to 2001) in the Metropolitan Detroit Cancer Surveillance System. J Clin Oncol. 2004;22(14):2865–2872. 15254054 10.1200/JCO.2004.12.149

[3] Nayak L, Lee EQ, Wen PY. Epidemiology of brain metastases. Curr Oncol Rep. 2012;14(1):48–54. 22012633 10.1007/s11912-011-0203-y

[4] Tsukada Y, Fouad A, Pickren JW, Lane WW. Central nervous system metastasis from breast carcinoma. Autopsy study. Cancer. 1983;52(12):2349–2354. 6640506 10.1002/1097-0142(19831215)52:12<2349::aid-cncr2820521231>3.0.co;2-b

[5] Neman J, Franklin M, Madaj Z, et al Use of predictive spatial modeling to reveal that primary cancers have distinct central nervous system topography patterns of brain metastasis. J Neurosurg. Published online July 16, 2021:1–9. 10.3171/2021.1.JNS203536 PMC8824486 34271545

[6] Liu Y, Cao X. Characteristics and significance of the pre-metastatic niche. Cancer Cell. 2016;30(5):668–681. 27846389 10.1016/j.ccell.2016.09.011

[7] Zou Y, Watters A, Cheng N, et al. Polyunsaturated fatty acids from astrocytes activate PPARγ signaling in cancer cells to promote brain metastasis. Cancer Discov. 2019;9(12):1720–1735. 31578185 10.1158/2159-8290.CD-19-0270 PMC6891206

[8] Medeiros B, Allan AL. Molecular mechanisms of breast cancer metastasis to the lung: clinical and experimental perspectives. Int J Mol Sci. 2019;20(9): 2272. 10.3390/ijms20092272 PMC6540248 31071959

[9] Wang D, Sun H, Wei J, Cen B, DuBois RN. CXCL1 is critical for premetastatic niche formation and metastasis in colorectal cancer. Cancer Res. 2017;77(13):3655–3665. 28455419 10.1158/0008-5472.CAN-16-3199 PMC5877403

[10] Salvador F, Martin A, López-Menéndez C, et al. Lysyl oxidase-like protein LOXL2 promotes lung metastasis of breast cancer. Cancer Res. 2017;77(21):5846–5859. 28720577 10.1158/0008-5472.CAN-16-3152 PMC5656180

[11] Qian BZ, Li J, Zhang H, et al. CCL2 recruits inflammatory monocytes to facilitate breast-tumour metastasis. Nature. 2011;475(7355):222–225. 21654748 10.1038/nature10138 PMC3208506

[12] Feng S, Cen J, Huang Y, et al. Matrix metalloproteinase-2 and -9 secreted by leukemic cells increase the permeability of blood-brain barrier by disrupting tight junction proteins. PLoS One. 2011;6(8):e20599. 21857898 10.1371/journal.pone.0020599 PMC3157343

[13] Feng S, Huang Y, Chen Z. Does VEGF secreted by leukemic cells increase the permeability of blood-brain barrier by disrupting tight-junction proteins in central nervous system leukemia? Med Hypotheses. 2011;76(5):618–621. 21398042 10.1016/j.mehy.2010.12.001

[14] Zhang HT, Zhang P, Gao Y, et al. Early VEGF inhibition attenuates blood-brain barrier disruption in ischemic rat brains by regulating the expression of MMPs. Mol Med Rep. 2017;15(1):57–64. 27909732 10.3892/mmr.2016.5974 PMC5355683

[15] Li B, Wang C, Zhang Y, et al. Elevated PLGF contributes to small-cell lung cancer brain metastasis. Oncogene. 2013;32(24):2952–2962. 22797069 10.1038/onc.2012.313

[16] Lyle LT, Lockman PR, Adkins CE, et al. Alterations in pericyte subpopulations are associated with elevated blood-tumor barrier permeability in experimental brain metastasis of breast cancer. Clin Cancer Res. 2016;22(21):5287–5299. 27245829 10.1158/1078-0432.CCR-15-1836 PMC5093086

[17] Soto MS, Serres S, Anthony DC, Sibson NR. Functional role of endothelial adhesion molecules in the early stages of brain metastasis. Neuro Oncol. 2014;16(4):540–551. 24311639 10.1093/neuonc/not222 PMC3956349

[18] Morad G, Carman CV, Hagedorn EJ, et al. Tumor-derived extracellular vesicles breach the intact blood-brain barrier via transcytosis. ACS Nano. 2019;13(12):13853–13865. 31479239 10.1021/acsnano.9b04397 PMC7169949

[19] Fong MY, Zhou W, Liu L, et al. Breast-cancer-secreted miR-122 reprograms glucose metabolism in premetastatic niche to promote metastasis. Nat Cell Biol. 2015;17(2):183–194. 25621950 10.1038/ncb3094 PMC4380143

[20] Tominaga N, Kosaka N, Ono M, et al. Brain metastatic cancer cells release microRNA-181c-containing extracellular vesicles capable of destructing blood-brain barrier. Nat Commun. 2015; 6:6716. 25828099 10.1038/ncomms7716 PMC4396394

[21] Rodrigues G, Hoshino A, Kenific CM, et al. Tumour exosomal CEMIP protein promotes cancer cell colonization in brain metastasis. Nat Cell Biol. 2019;21(11):1403–1412. 31685984 10.1038/s41556-019-0404-4 PMC7354005

[22] Berghoff AS, Liao Y, Karreman MA, et al. Identification and characterization of cancer cells that initiate metastases to the brain and other organs. Mol Cancer Res. 2021;19(4):688–701. 33443114 10.1158/1541-7786.MCR-20-0863 PMC9281611

[23] Boral D, Vishnoi M, Liu HN, et al. Molecular characterization of breast cancer CTCs associated with brain metastasis. Nat Commun. 2017;8(1):196. 28775303 10.1038/s41467-017-00196-1 PMC5543046

[24] Singh M, Venugopal C, Tokar T, et al. Therapeutic targeting of the premetastatic stage in human lung-to-brain metastasis. Cancer Res. 2018;78(17):5124–5134. 29986997 10.1158/0008-5472.CAN-18-1022

[25] Ren D, Zhu X, Kong R, et al. Targeting brain-adaptive cancer stem cells prohibits brain metastatic colonization of triple-negative breast cancer. Cancer Res. 2018;78(8):2052–2064. 29567857 10.1158/0008-5472.CAN-17-2994 PMC5899649

[26] Kienast Y, von Baumgarten L, Fuhrmann M, et al. Real-time imaging reveals the single steps of brain metastasis formation. Nat Med. 2010;16(1):116–122. 20023634 10.1038/nm.2072

[27] Winkler F . Pathogenesis and biology. Handb Clin Neurol. 2018;149:43–56. 29307360 10.1016/B978-0-12-811161-1.00003-7

[28] Yao H, Price TT, Cantelli G, et al. Leukaemia hijacks a neural mechanism to invade the central nervous system. Nature. 2018;560(7716):55–60. 30022166 10.1038/s41586-018-0342-5 PMC10257142

[29] Bovetti S, Hsieh YC, Bovolin P, Perroteau I, Kazunori T, Puche AC. Blood vessels form a scaffold for neuroblast migration in the adult olfactory bulb. J Neurosci. 2007;27(22):5976–5980. 17537968 10.1523/JNEUROSCI.0678-07.2007 PMC6672264

[30] Kokkoris CP . Leptomeningeal carcinomatosis. How does cancer reach the pia-arachnoid? Cancer. 1983;51(1):154–160. 6336971 10.1002/1097-0142(19830101)51:1<154::aid-cncr2820510130>3.0.co;2-k

[31] Witzel I, Oliveira-Ferrer L, Pantel K, Müller V, Wikman H. Breast cancer brain metastases: biology and new clinical perspectives. Breast Cancer Res. 2016;18(1):8. 26781299 10.1186/s13058-015-0665-1 PMC4717619

[32] Feinauer MJ, Schneider SW, Berghoff AS, et al. Local blood coagulation drives cancer cell arrest and brain metastasis in a mouse model. Blood. 2021;137(9):1219–1232. 33270819 10.1182/blood.2020005710

[33] Eyler CE, Foo WC, LaFiura KM, McLendon RE, Hjelmeland AB, Rich JN. Brain cancer stem cells display preferential sensitivity to Akt inhibition. Stem Cells. 2008;26(12):3027–3036. 18802038 10.1634/stemcells.2007-1073 PMC2739007

[34] Wang X, Prager BC, Wu Q, et al. Reciprocal signaling between glioblastoma stem cells and differentiated tumor cells promotes malignant progression. Cell Stem Cell. 2018;22(4):514–528.e5. 29625067 10.1016/j.stem.2018.03.011 PMC5947947

[35] Ricci-Vitiani L, Pallini R, Biffoni M, et al. Tumour vascularization via endothelial differentiation of glioblastoma stem-like cells. Nature. 2010;468(7325):824–828. 21102434 10.1038/nature09557

[36] Charles N, Holland EC. The perivascular niche microenvironment in brain tumor progression. Cell Cycle. 2010;9(15):3084–3093. 10.4161/cc.9.15.12710 PMC3040926 20714216

[37] Calabrese C, Poppleton H, Kocak M, et al. A perivascular niche for brain tumor stem cells. Cancer Cell. 2007;11(1):69–82. 17222791 10.1016/j.ccr.2006.11.020

[38] Infanger DW, Cho Y, Lopez BS, et al. Glioblastoma stem cells are regulated by interleukin-8 signaling in a tumoral perivascular niche. Cancer Res. 2013;73(23):7079–7089. 24121485 10.1158/0008-5472.CAN-13-1355 PMC3880850

[39] Wang Z, Zhang H, Xu S, Liu Z, Cheng Q. The adaptive transition of glioblastoma stem cells and its implications on treatments. Sig Transduct Target Ther. 2021;6(1):1–13. 10.1038/s41392-021-00491-w PMC7985200 33753720

[40] Berghoff AS, Rajky O, Winkler F, et al. Invasion patterns in brain metastases of solid cancers. Neuro Oncol. 2013;15(12):1664–1672. 24084410 10.1093/neuonc/not112 PMC3829586

[41] Er EE, Valiente M, Ganesh K, et al. Pericyte-like spreading by disseminated cancer cells activates YAP and MRTF for metastatic colonization. Nat Cell Biol. 2018;20(8):966–978. 30038252 10.1038/s41556-018-0138-8 PMC6467203

[42] Carbonell WS, Ansorge O, Sibson N, Muschel R. The vascular basement membrane as “soil” in brain metastasis. PLoS One. 2009;4(6):e5857. 19516901 10.1371/journal.pone.0005857 PMC2689678

[43] Valiente M, Obenauf AC, Jin X, et al. Serpins promote cancer cell survival and vascular co-option in brain metastasis. Cell. 2014;156(5):1002–1016. 24581498 10.1016/j.cell.2014.01.040 PMC3988473

[44] Jubb AM, Cesario A, Ferguson M, et al. Vascular phenotypes in primary non-small cell lung carcinomas and matched brain metastases. Br J Cancer. 2011;104(12):1877–1881. 21540863 10.1038/bjc.2011.147 PMC3111192

[45] Carmeliet P . VEGF as a key mediator of angiogenesis in cancer. Oncology. 2005;69(suppl 3):4–10. 16301830 10.1159/000088478

[46] Lorger M, Krueger JS, O’Neal M, Staflin K, Felding-Habermann B. Activation of tumor cell integrin alphavbeta3 controls angiogenesis and metastatic growth in the brain. Proc Natl Acad Sci U S A. 2009;106(26):10666–10671. 19541645 10.1073/pnas.0903035106 PMC2697113

[47] Kim LS, Huang S, Lu W, Lev DC, Price JE. Vascular endothelial growth factor expression promotes the growth of breast cancer brain metastases in nude mice. Clin Exp Metastasis. 2004;21(2):107–118. 15168728 10.1023/b:clin.0000024761.00373.55

[48] Yano S, Shinohara H, Herbst RS, et al. Expression of vascular endothelial growth factor is necessary but not sufficient for production and growth of brain metastasis. Cancer Res. 2000;60(17):4959–4967. 10987313

[49] Bohn KA, Adkins CE, Nounou MI, Lockman PR. Inhibition of VEGF and angiopoietin-2 to reduce brain metastases of breast cancer burden. Front Pharmacol. 2017;8:193. 28443023 10.3389/fphar.2017.00193 PMC5387068

[50] Ilhan-Mutlu A, Osswald M, Liao Y, et al. Bevacizumab prevents brain metastases formation in lung adenocarcinoma. Mol Cancer Ther. 2016;15(4):702–710. 26809491 10.1158/1535-7163.MCT-15-0582

[51] Engelhardt B, Sorokin L. The blood-brain and the blood-cerebrospinal fluid barriers: function and dysfunction. Semin Immunopathol. 2009;31(4):497–511. 19779720 10.1007/s00281-009-0177-0

[52] Daneman R, Prat A. The blood-brain barrier. Cold Spring Harb Perspect Biol. 2015;7(1):a020412. 25561720 10.1101/cshperspect.a020412 PMC4292164

[53] Kadry H, Noorani B, Cucullo L. A blood-brain barrier overview on structure, function, impairment, and biomarkers of integrity. Fluids Barriers CNS. 2020;17(1):69. 33208141 10.1186/s12987-020-00230-3 PMC7672931

[54] Zlokovic BV . The blood-brain barrier in health and chronic neurodegenerative disorders. Neuron. 2008;57(2):178–201. 18215617 10.1016/j.neuron.2008.01.003

[55] Lorger M, Felding-Habermann B. Capturing changes in the brain microenvironment during initial steps of breast cancer brain metastasis. Am J Pathol. 2010;176(6):2958–2971. 20382702 10.2353/ajpath.2010.090838 PMC2877856

[56] Yonemori K, Tsuta K, Ono M, et al. Disruption of the blood brain barrier by brain metastases of triple-negative and basal-type breast cancer but not HER2/neu-positive breast cancer. Cancer. 2010;116(2):302–308. 19937674 10.1002/cncr.24735

[57] Fazakas C, Wilhelm I, Nagyoszi P, et al. Transmigration of melanoma cells through the blood-brain barrier: role of endothelial tight junctions and melanoma-released serine proteases. PLoS One. 2011;6(6):e20758. 21674054 10.1371/journal.pone.0020758 PMC3107231

[58] Klemke M, Weschenfelder T, Konstandin MH, Samstag Y. High affinity interaction of integrin alpha4beta1 (VLA-4) and vascular cell adhesion molecule 1 (VCAM-1) enhances migration of human melanoma cells across activated endothelial cell layers. J Cell Physiol. 2007;212(2):368–374. 17352405 10.1002/jcp.21029

[59] Tiwary S, Morales JE, Kwiatkowski SC, Lang FF, Rao G, McCarty JH. Metastatic brain tumors disrupt the blood-brain barrier and alter lipid metabolism by inhibiting expression of the endothelial cell fatty acid transporter Mfsd2a. Sci Rep. 2018;8(1):8267. 29844613 10.1038/s41598-018-26636-6 PMC5974340

[60] Bos PD, Zhang XH, Nadal C, et al. Genes that mediate breast cancer metastasis to the brain. Nature. 2009;459(7249):1005–1009. 19421193 10.1038/nature08021 PMC2698953

[61] Hu L, Zhang J, Zhu H, Min J, Feng Y, Zhang H. Biological characteristics of a specific brain metastatic cell line derived from human lung adenocarcinoma. Med Oncol. 2010;27(3):708–714. 19669609 10.1007/s12032-009-9273-1

[62] Nag S , ed. The Blood-Brain and Other Neural Barriers: Reviews and Protocols. Totowa, NJ: Humana Press; 2011.

[63] Redzic Z . Molecular biology of the blood-brain and the blood-cerebrospinal fluid barriers: similarities and differences. Fluids Barriers CNS. 2011;8(1):3. 21349151 10.1186/2045-8118-8-3 PMC3045361

[64] Deshpande K, Buchanan I, Martirosian V, Neman J. Clinical perspectives in brain metastasis. Cold Spring Harb Perspect Med. 2020;10(6). 10.1101/cshperspect.a037051 PMC7263089 31615863

[65] Boire A, Zou Y, Shieh J, Macalinao DG, Pentsova E, Massagué J. Complement component 3 adapts the cerebrospinal fluid for leptomeningeal metastasis. Cell. 2017;168(6):1101–1113.e13. 28283064 10.1016/j.cell.2017.02.025 PMC5405733

[66] Herrera RA, Deshpande K, Martirosian V, et al Cortisol promotes breast-to-brain metastasis through the blood-cerebrospinal fluid barrier. Cancer Rep (Hoboken). Published online February 26, 2021:e1351. 33635590 10.1002/cnr2.1351 PMC9124512

[67] Vandenhaute E, Stump-Guthier C, Lasierra Losada M, et al. The choroid plexus may be an underestimated site of tumor invasion to the brain: an in vitro study using neuroblastoma cell lines. Cancer Cell Int. 2015;15:102. 26500454 10.1186/s12935-015-0257-2 PMC4619509

[68] Schild T, Low V, Blenis J, Gomes AP. Unique metabolic adaptations dictate distal organ-specific metastatic colonization. Cancer Cell. 2018;33(3):347–354. 29533780 10.1016/j.ccell.2018.02.001 PMC5889305

[69] Chen J, Lee HJ, Wu X, et al. Gain of glucose-independent growth upon metastasis of breast cancer cells to the brain. Cancer Res. 2015;75(3):554–565. 25511375 10.1158/0008-5472.CAN-14-2268 PMC4315743

[70] Mashimo T, Pichumani K, Vemireddy V, et al. Acetate is a bioenergetic substrate for human glioblastoma and brain metastases. Cell. 2014;159(7):1603–1614. 25525878 10.1016/j.cell.2014.11.025 PMC4374602

[71] Fischer GM, Jalali A, Kircher DA, et al. Molecular profiling reveals unique immune and metabolic features of melanoma brain metastases. Cancer Discov. 2019;9(5):628–645. 30787016 10.1158/2159-8290.CD-18-1489 PMC6497554

[72] Fischer GM, Guerrieri RA, Hu Q, et al. Clinical, molecular, metabolic, and immune features associated with oxidative phosphorylation in melanoma brain metastases. Neurooncol Adv. 2021;3(1):vdaa177. 33575655 10.1093/noajnl/vdaa177 PMC7865080

[73] Fukumura K, Malgulwar PB, Fischer GM, et al. Multi-omic molecular profiling reveals potentially targetable abnormalities shared across multiple histologies of brain metastasis. Acta Neuropathol. 2021;141(2):303–321. 33394124 10.1007/s00401-020-02256-1 PMC7852029

[74] Haq R, Shoag J, Andreu-Perez P, et al. Oncogenic BRAF regulates oxidative metabolism via PGC1α and MITF. Cancer Cell. 2013;23(3):302–315. 23477830 10.1016/j.ccr.2013.02.003 PMC3635826

[75] Ebright RY, Zachariah MA, Micalizzi DS, et al. HIF1A signaling selectively supports proliferation of breast cancer in the brain. Nat Commun. 2020;11(1):6311. 33298946 10.1038/s41467-020-20144-w PMC7725834

[76] Ngo B, Kim E, Osorio-Vasquez V, et al. Limited environmental serine and glycine confer brain metastasis sensitivity to PHGDH inhibition. Cancer Discov. 2020;10(9):1352–1373. 32571778 10.1158/2159-8290.CD-19-1228 PMC7483776

[77] Sofroniew MV, Vinters HV. Astrocytes: biology and pathology. Acta Neuropathol. 2010;119(1):7–35. 20012068 10.1007/s00401-009-0619-8 PMC2799634

[78] Placone AL, Quiñones-Hinojosa A, Searson PC. The role of astrocytes in the progression of brain cancer: complicating the picture of the tumor microenvironment. Tumour Biol. 2016;37(1):61–69. 26493995 10.1007/s13277-015-4242-0

[79] Samdani AF, Kuchner EB, Rhines L, et al. Astroglia induce cytotoxic effects on brain tumors via a nitric oxide-dependent pathway both in vitro and in vivo. Neurosurgery. 2004;54(5):1231–7; discussion 1237. 15113479 10.1227/01.neu.0000119576.76193.b8

[80] Seike T, Fujita K, Yamakawa Y, et al. Interaction between lung cancer cells and astrocytes via specific inflammatory cytokines in the microenvironment of brain metastasis. Clin Exp Metastasis. 2011;28(1):13–25. 20953899 10.1007/s10585-010-9354-8 PMC2998640

[81] Chen Q, Boire A, Jin X, et al. Carcinoma-astrocyte gap junctions promote brain metastasis by cGAMP transfer. Nature. 2016;533(7604):493–498. 27225120 10.1038/nature18268 PMC5021195

[82] Lin Q, Balasubramanian K, Fan D, et al. Reactive astrocytes protect melanoma cells from chemotherapy by sequestering intracellular calcium through gap junction communication channels. Neoplasia. 2010;12(9):748–754. 20824051 10.1593/neo.10602 PMC2933695

[83] Marchetti D, Li J, Shen R. Astrocytes contribute to the brain-metastatic specificity of melanoma cells by producing heparanase. Cancer Res. 2000;60(17):4767–4770. 10987284

[84] Boukerche H, Su ZZ, Kang DC, Fisher PB. Identification and cloning of genes displaying elevated expression as a consequence of metastatic progression in human melanoma cells by rapid subtraction hybridization. Gene. 2004;343(1):191–201. 15563845 10.1016/j.gene.2004.09.002

[85] Langley RR, Fan D, Guo L, et al. Generation of an immortalized astrocyte cell line from H-2Kb-tsA58 mice to study the role of astrocytes in brain metastasis. Int J Oncol. 2009;35(4):665–672. 19724901 10.3892/ijo_00000378 PMC2882684

[86] Kim SJ, Kim JS, Park ES, et al. Astrocytes upregulate survival genes in tumor cells and induce protection from chemotherapy. Neoplasia. 2011;13(3):286–298. 21390191 10.1593/neo.11112 PMC3050871

[87] Priego N, Zhu L, Monteiro C, et al. STAT3 labels a subpopulation of reactive astrocytes required for brain metastasis. Nat Med. 2018;24(7):1024–1035. 29892069 10.1038/s41591-018-0044-4

[88] Seano G, Nia HT, Emblem KE, et al. Solid stress in brain tumours causes neuronal loss and neurological dysfunction and can be reversed by lithium. Nat Biomed Eng. 2019;3(3):230–245. 30948807 10.1038/s41551-018-0334-7 PMC6452896

[89] Fitzgerald DP, Subramanian P, Deshpande M, et al. Opposing effects of pigment epithelium-derived factor on breast cancer cell versus neuronal survival: implication for brain metastasis and metastasis-induced brain damage. Cancer Res. 2012;72(1):144–153. 22215693 10.1158/0008-5472.CAN-11-1904 PMC3254209

[90] Neman J, Termini J, Wilczynski S, et al. Human breast cancer metastases to the brain display GABAergic properties in the neural niche. Proc Natl Acad Sci U S A. 2014;111(3):984–989. 24395782 10.1073/pnas.1322098111 PMC3903266

[91] Termini J, Neman J, Jandial R. Role of the neural niche in brain metastatic cancer. Cancer Res. 2014;74(15):4011–4015. 25035392 10.1158/0008-5472.CAN-14-1226 PMC4122250

[92] Zeng Q, Michael IP, Zhang P, et al. Synaptic proximity enables NMDAR signalling to promote brain metastasis. Nature. 2019;573(7775):526–531. 31534217 10.1038/s41586-019-1576-6 PMC6837873

[93] Jordan FL, Thomas WE. Brain macrophages: questions of origin and interrelationship. Brain Res. 1988;472(2):165–178. 3289689 10.1016/0165-0173(88)90019-7

[94] You H, Baluszek S, Kaminska B. Supportive roles of brain macrophages in CNS metastases and assessment of new approaches targeting their functions. Theranostics. 2020;10(7):2949–2964. 32194848 10.7150/thno.40783 PMC7053204

[95] Rippaus N, Taggart D, Williams J, et al. Metastatic site-specific polarization of macrophages in intracranial breast cancer metastases. Oncotarget. 2016;7(27):41473–41487. 27203741 10.18632/oncotarget.9445 PMC5173073

[96] Pukrop T, Dehghani F, Chuang HN, et al. Microglia promote colonization of brain tissue by breast cancer cells in a Wnt-dependent way. Glia. 2010;58(12):1477–1489. 20549749 10.1002/glia.21022

[97] Bechmann I, Priller J, Kovac A, et al. Immune surveillance of mouse brain perivascular spaces by blood-borne macrophages. Eur J Neurosci. 2001;14(10):1651–1658. 11860459 10.1046/j.0953-816x.2001.01793.x

[98] Friebel E, Kapolou K, Unger S, et al. Single-cell mapping of human brain cancer reveals tumor-specific instruction of tissue-invading leukocytes. Cell. 2020;181(7):1626–1642.e20. 32470397 10.1016/j.cell.2020.04.055

[99] Chaudhary B, Elkord E. Regulatory T cells in the tumor microenvironment and cancer progression: role and therapeutic targeting. Vaccines (Basel). 2016;4(3). 10.3390/vaccines4030028 PMC5041022 27509527

[100] Woroniecka K, Fecci PE. T-cell exhaustion in glioblastoma. Oncotarget. 2018;9(82):35287–35288. 30450155 10.18632/oncotarget.26228 PMC6219672

[101] Gill CM, D’Andrea MR, Tomita S, et al Tumor immune microenvironment in brain metastases from gynecologic malignancies. Cancer Immunol Immunother. Published online March 13, 2021. 10.1007/s00262-021-02909-4 PMC10992931 33713153

[102] Harter PN, Bernatz S, Scholz A, et al. Distribution and prognostic relevance of tumor-infiltrating lymphocytes (TILs) and PD-1/PD-L1 immune checkpoints in human brain metastases. Oncotarget. 2015;6(38):40836–40849. 26517811 10.18632/oncotarget.5696 PMC4747372

[103] Ogiya R, Niikura N, Kumaki N, et al. Comparison of immune microenvironments between primary tumors and brain metastases in patients with breast cancer. Oncotarget. 2017;8(61):103671–103681. 29262592 10.18632/oncotarget.22110 PMC5732758

[104] Song E, Mao T, Dong H, et al. VEGF-C-driven lymphatic drainage enables immunosurveillance of brain tumours. Nature. 2020;577(7792):689–694. 31942068 10.1038/s41586-019-1912-x PMC7100608

[105] Klemm F, Maas RR, Bowman RL, et al. Interrogation of the microenvironmental landscape in brain tumors reveals disease-specific alterations of immune cells. Cell. 2020;181(7):1643–1660.e17. 32470396 10.1016/j.cell.2020.05.007 PMC8558904

[106] Yang TH, St John LS, Garber HR, et al. Membrane-associated proteinase 3 on granulocytes and acute myeloid leukemia inhibits T cell proliferation. J Immunol. 2018;201(5):1389–1399. 30021768 10.4049/jimmunol.1800324 PMC6099529

[107] Berghoff AS, Fuchs E, Ricken G, et al. Density of tumor-infiltrating lymphocytes correlates with extent of brain edema and overall survival time in patients with brain metastases. Oncoimmunology. 2016;5(1):e1057388. 26942067 10.1080/2162402X.2015.1057388 PMC4760339

[108] Chongsathidkiet P, Jackson C, Koyama S, et al. Sequestration of T cells in bone marrow in the setting of glioblastoma and other intracranial tumors. Nat Med. 2018;24(9):1459–1468. 30104766 10.1038/s41591-018-0135-2 PMC6129206

[109] Kovalchuk B, Berghoff AS, Karreman MA, et al. Nintedanib and a bi-specific anti-VEGF/Ang2 nanobody selectively prevent brain metastases of lung adenocarcinoma cells. Clin Exp Metastasis. 2020;37(6):637–648. 32918638 10.1007/s10585-020-10055-x PMC7666285

[110] Berghoff AS, Breckwoldt MO, Riedemann L, et al. Bevacizumab-based treatment as salvage therapy in patients with recurrent symptomatic brain metastases. Neurooncol Adv. 2020;2(1):vdaa038. 32642693 10.1093/noajnl/vdaa038 PMC7212911

[111] Besse B, Le Moulec S, Mazières J, et al. Bevacizumab in patients with nonsquamous non-small cell lung cancer and asymptomatic, untreated brain metastases (BRAIN): a nonrandomized, phase II study. Clin Cancer Res. 2015;21(8):1896–1903. 25614446 10.1158/1078-0432.CCR-14-2082

[112] Lu YS, Chen TW, Lin CH, et al.; Taiwan Breast Cancer Consortium. Bevacizumab preconditioning followed by etoposide and cisplatin is highly effective in treating brain metastases of breast cancer progressing from whole-brain radiotherapy. Clin Cancer Res. 2015;21(8):1851–1858. 25700303 10.1158/1078-0432.CCR-14-2075

[113] Fecci PE, Champion CD, Hoj J, et al. The evolving modern management of brain metastasis. Clin Cancer Res. 2019;25(22):6570–6580. 31213459 10.1158/1078-0432.CCR-18-1624 PMC8258430

[114] Khan M, Lin J, Liao G, et al. Comparison of WBRT alone, SRS alone, and their combination in the treatment of one or more brain metastases: review and meta-analysis. Tumour Biol. 2017;39(7):1010428317702903. 28675121 10.1177/1010428317702903

[115] Brown PD, Brown CA, Pollock BE, Gorman DA, Foote RL. Stereotactic radiosurgery for patients with “radioresistant” brain metastases. Neurosurgery. 2002;51(3):656–65; discussion 665. 12188943

[116] Okimoto T, Tsubata Y, Hotta T, et al. A low crizotinib concentration in the cerebrospinal fluid causes ineffective treatment of anaplastic lymphoma kinase-positive non-small cell lung cancer with carcinomatous meningitis. Intern Med. 2019;58(5):703–705. 30333394 10.2169/internalmedicine.1072-18 PMC6443566

[117] Agarwal S, Sane R, Gallardo JL, Ohlfest JR, Elmquist WF. Distribution of gefitinib to the brain is limited by P-glycoprotein (ABCB1) and breast cancer resistance protein (ABCG2)-mediated active efflux. J Pharmacol Exp Ther. 2010;334(1):147–155. 20421331 10.1124/jpet.110.167601 PMC2912048

[118] Lagas JS, van Waterschoot RA, Sparidans RW, Wagenaar E, Beijnen JH, Schinkel AH. Breast cancer resistance protein and P-glycoprotein limit sorafenib brain accumulation. Mol Cancer Ther. 2010;9(2):319–326. 20103600 10.1158/1535-7163.MCT-09-0663

[119] Fidler IJ . The role of the organ microenvironment in brain metastasis. Semin Cancer Biol. 2011;21(2):107–112. 21167939 10.1016/j.semcancer.2010.12.009

[120] Niessner H, Forschner A, Klumpp B, et al. Targeting hyperactivation of the AKT survival pathway to overcome therapy resistance of melanoma brain metastases. Cancer Med. 2013;2(1):76–85. 24133630 10.1002/cam4.50 PMC3797558

[121] Kodack DP, Askoxylakis V, Ferraro GB, et al The brain microenvironment mediates resistance in luminal breast cancer to PI3K inhibition through HER3 activation. Sci Transl Med. 2017;9(391). 10.1126/scitranslmed.aal4682 PMC5917603 28539475

[122] Alečković M, Kang Y. Welcoming treat: astrocyte-derived exosomes induce PTEN suppression to foster brain metastasis. Cancer Cell. 2015;28(5):554–556. 26555172 10.1016/j.ccell.2015.10.010

[123] Bachelot T, Romieu G, Campone M, et al. Lapatinib plus capecitabine in patients with previously untreated brain metastases from HER2-positive metastatic breast cancer (LANDSCAPE): a single-group phase 2 study. Lancet Oncol. 2013;14(1):64–71. 23122784 10.1016/S1470-2045(12)70432-1

[124] Davies MA, Saiag P, Robert C, et al. Dabrafenib plus trametinib in patients with BRAFV600-mutant melanoma brain metastases (COMBI-MB): a multicentre, multicohort, open-label, phase 2 trial. Lancet Oncol. 2017;18(7):863–873. 28592387 10.1016/S1470-2045(17)30429-1 PMC5991615

[125] Freedman RA, Gelman RS, Anders CK, et al.; Translational Breast Cancer Research Consortium. TBCRC 022: a phase II trial of neratinib and capecitabine for patients with human epidermal growth factor receptor 2-positive breast cancer and brain metastases. J Clin Oncol. 2019;37(13):1081–1089. 30860945 10.1200/JCO.18.01511 PMC6494354

[126] Goldberg SB, Gettinger SN, Mahajan A, et al. Pembrolizumab for patients with melanoma or non-small-cell lung cancer and untreated brain metastases: early analysis of a non-randomised, open-label, phase 2 trial. Lancet Oncol. 2016;17(7):976–983. 27267608 10.1016/S1470-2045(16)30053-5 PMC5526047

[127] Amaral T, Tampouri I, Eigentler T, et al. Immunotherapy plus surgery/radiosurgery is associated with favorable survival in patients with melanoma brain metastasis. Immunotherapy. 2019;11(4):297–309. 30606066 10.2217/imt-2018-0149

[128] Kluger HM, Chiang V, Mahajan A, et al. Long-term survival of patients with melanoma with active brain metastases treated with pembrolizumab on a phase II trial. J Clin Oncol. 2019;37(1):52–60. 30407895 10.1200/JCO.18.00204 PMC6354772

[129] Margolin K, Ernstoff MS, Hamid O, et al. Ipilimumab in patients with melanoma and brain metastases: an open-label, phase 2 trial. Lancet Oncol. 2012;13(5):459–465. 22456429 10.1016/S1470-2045(12)70090-6

[130] Knisely JP, Yu JB, Flanigan J, Sznol M, Kluger HM, Chiang VL. Radiosurgery for melanoma brain metastases in the ipilimumab era and the possibility of longer survival. J Neurosurg. 2012;117(2):227–233. 22702482 10.3171/2012.5.JNS111929 PMC6098938

[131] Tehranian C, Fankhauser L, Harter PN, et al The PI3K/Akt/mTOR pathway as a preventive target in melanoma brain metastasis. Neuro Oncol. Published online July 3, 2021:noab159. doi:10.1093/neuonc/noab159 34216217 PMC8804893

[132] Zimmer AS, Steinberg SM, Smart DD, et al. Temozolomide in secondary prevention of HER2-positive breast cancer brain metastases. Future Oncol. 2020;16(14):899–909. 32270710 10.2217/fon-2020-0094 PMC7270957

